# Supplementary material for: Evaluating the impact of the nationwide public–private mix (PPM) program for tuberculosis under National Health Insurance in South Korea: A difference in differences analysis
Source: PLoS Med. 2021 Jul 14;18(7):e1003717. doi: 10.1371/journal.pmed.1003717 (PMC8318235; doi:10.1371/journal.pmed.1003717)
Supplement: S1 File — Fig A: Treatment outcome assessment criteria for patients with multiple registration records in a given year. Fig B: A trend of TS rate between public and private sectors in 1987, 1993, 2009, and 2014. Fig C: Distribution of propensity scores before and after kernel matching in 2009 and 2014. Table A: Compared analysis of DID with various methods of PSM. Table B: Subgroup analysis of DID with PSM on LTFU rate in public and private sectors in 2009 and 2014. Table C: Subgroup analysis of DID with PSM on TS rate in public and private sectors in 2009 and 2014. Table D: Comparing the results of DID after using categorical and continuous variable. Table E: Absolute and relative risk differences between public and private sectors in 2009 and 2014. DID, difference in differences; LTFU, loss to follow-up; PSM, propensity score matching; TS, treatment success. (DOCX) [file pmed.1003717.s001.docx]

**Supplementary Figures and Tables**

**Supplementary materials to manuscript:**

**Evaluating the Impact of the Nationwide Public-Private Mix (PPM) Program for Tuberculosis under National Health Insurance in South Korea: A Difference in Differences Analysis**

Sarah Yu^1,3,4†^, Hojoon Sohn^2†^, Hae-Young Kim^3,4^, Hyunwoo Kim^1,5^, Kyung-Hyun Oh^1,6^, Hee-Jin Kim^1^, Haejoo Chung^3,4^, Hongjo Choi^1,7*^

† Contributed equally to this manuscript

**Author Affiliations**

1. Korean Institute of Tuberculosis, Korean National Tuberculosis Association, Cheongju, Republic of Korea
2. Department of Epidemiology, Johns Hopkins Bloomberg School of Public Health, Baltimore, Maryland, USA
3. School of Health Policy & Management, College of Health Science, Korea University, Seoul, Republic of Korea
4. BK21 FOUR R&E Center for Learning Health Systems, Korea University, Seoul, Republic of Korea
5. Department of Health Research Methods, Evidence, and Impact, McMaster University, Hamilton, Ontario, Canada
6. End TB and Leprosy Unit, World Health Organization Regional Office for the Western Pacific, Manila, Philippines
7. Department of Preventive Medicine, College of Medicine, Konyang University, Daejeon, Republic of Korea

Contents

Assessment of Treatment Outcomes1

Fig A. Treatment outcome assessment criteria for patients with multiple registration records in a given year. 1

Parallel Trends Assumption2

Fig B. A trend of treatment success rate between public and private sectors in 1987, 1993, 2009 and 2014 2

Propensity Score Matching4

Fig C. Distribution of propensity scores before and after kernel matching in 2009 and 2014 4

Table A. Compared analysis of Difference in differences with various methods of propensity score marching 5

Subgroup analysis in Difference in Differences6

Table B. Subgroup analysis of difference in differences with propensity score matching on loss to follow-up rate in public and private sectors in 2009 and 2014 6

Table C. Subgroup analysis of difference in differences with propensity score matching on treatment success rate in public and private sectors in 2009 and 2014 8

Categorical or Continuous variable9

Table D. Comparing the results of DID after using categorical and continuous variable 9

Absolute or Relative Risk10

Table E. Absolute and relative risk differences between public and private sectors in 2009 and 2014 10

**[Assessment of Treatment Outcomes in patients with multiple treatment registration in a given year]**


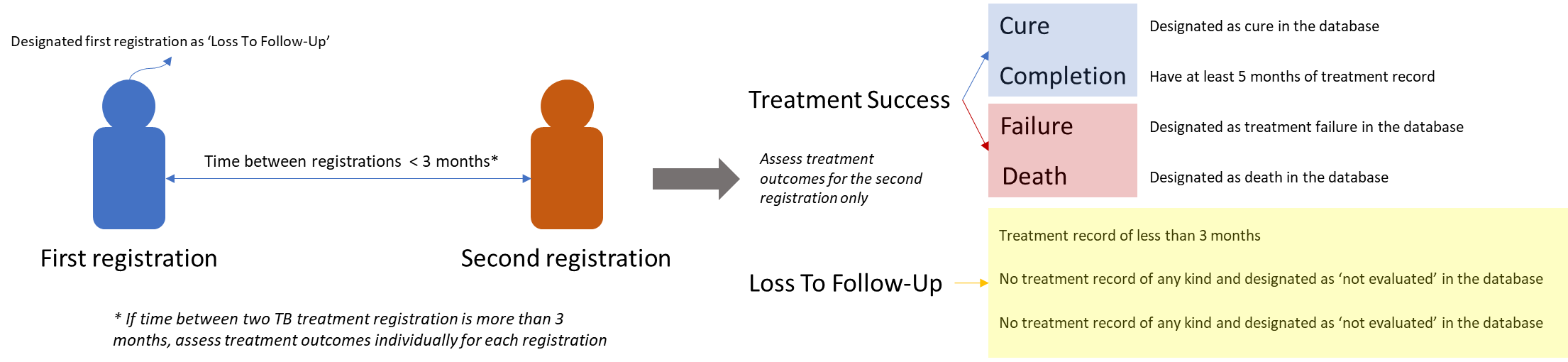


Fig A. Treatment outcome assessment criteria for patients with multiple registration records in a given year.

[**Parallel Trends Assumption]**

Fig B. A trend of treatment success rate between public and private sectors in 1987, 1993, 2009 and 2014.

The reference of public and private sector in 1987 and 1993: Hong YP, Kim SJ, Lee EG, Lew WJ, Bai JY. Treatment of bacillary pulmonary tuberculosis at the chest clinics in the private sector in Korea, 1993. International Journal of Tuberculosis and Lung Disease. 1999;3(8):695-702; The outcome of 2009 and 2014 was the study results.

|  |  |
| --- | --- |
|  |  |
| **Fig C. Distribution of propensity scores before and after kernel matching in 2009 and 2014** | |

[Propensity Score Matching]

Table A Compared analysis of Difference in differences with various methods of propensity score marching

|  | 1 to 1 (n=59,116) | | 1 to 3 (n=61,313) | | Kernel, rcs (n=66,382) | |
| --- | --- | --- | --- | --- | --- | --- |
|  | DID | t (p-value) | DID | t (p-value) | DID | t (p-value) |
| LTF | -0.088 | 4.89*** | -0.108 | 8.84*** | -0.087 | 17.21*** |
| TS | 0.052 | 2.48* | 0.068 | 4.81*** | 0.041 | 7.20*** |
| *p-value <0.05, ** p-value<0.01, *** p-value<0.001 | | | | | |  |

[Subgroup analysis in Difference in Differences]

Table B Subgroup analysis of difference in differences with propensity score matching on loss to follow-up rate in public and private sectors in 2009 and 2014

|  | | Public | | Private | | Crude | | | Kernel (PMS) | |
| --- | --- | --- | --- | --- | --- | --- | --- | --- | --- | --- |
| Risk factors | | 2009  (n=6,195) | 2014  (n=2,803) | 2009  (n=27,396) | 2014  (n=29,988) |  |  |  |  |  |
|  |  | N (%) | N (%) | N (%) | N (%) | DID | (95% CI) | | DID | (95%CI) |
| Overall |  | 701 (11.3) | 149 (5.3) | 6,904 (25.2) | 2,195 (7.3) | -0.119 | (-0.135, | -0.103) | -0.087 | (-0.0968, -0.0772) |
| Gender | Male | 481 (12.3) | 95 (5.4) | 4,099 (26.7) | 1,364 (8.1) | -0.117 | (-0.139, | -0.095) | -0.078 | (-0.0917, -0.0643) |
|  | Female | 220 (9.6) | 54 (5.2) | 2,805 (23.3) | 831 (6.4) | -0.126 | (-0.151, | -0.101) | -0.099 | (-0.1127, -0.0853) |
| Age group | -19 | 55 (6.0) | 9 (3.6) | 298 (20.3) | 49 (5.0) | -0.129 | (-0.178, | -0.080) | -0.154 | (-0.1932, -0.1148) |
|  | 20-29 | 126 (10.2) | 36 (6.8) | 892 (21.2) | 265 (7.7) | -0.101 | (-0.138, | -0.064) | -0.070 | (-0.0974, -0.0426) |
|  | 30-39 | 110 (11.2) | 15 (4.2) | 858 (21.8) | 210 (6.2) | -0.085 | (-0.128, | -0.042) | -0.076 | (-0.1015, -0.0505) |
|  | 40-49 | 131 (13.8) | 32 (7.2) | 1,082 (26.3) | 338 (8.0) | -0.116 | (-0.159, | -0.073) | -0.069 | (-0.0964, -0.0416) |
|  | 50-59 | 97 (13.0) | 27 (5.5) | 976 (25.8) | 385 (7.6) | -0.107 | (-0.148, | -0.066) | -0.128 | (-0.1535, -0.1025) |
|  | 60-69 | 72 (13.1) | 10 (3.4) | 972 (26.5) | 239 (6.2) | -0.106 | (-0.157, | -0.055) | -0.051 | (-0.0784, -0.0236) |
|  | 70- | 110 (13.5) | 20 (4.6) | 1,826 (29.4) | 709 (7.8) | -0.127 | (-0.170, | -0.084) | -0.114 | (-0.1336, -0.0944) |
| Nationality | Korean | 633 (10.9) | 104 (4.1) | 6,475 (24.6) | 1,925 (6.7) | -0.111 | (-0.129, | -0.093) | -0.083 | (-0.0928, -0.0732) |
|  | Others | 68 (18.8) | 45 (17.9) | 429 (40.7) | 270 (23.6) | -0.162 | (-0.242, | -0.082) | -0.223 | (-0.2877, -0.1583) |
| Transfer history | Treated at 1 site | 378 (8.0) | 95 (4.3) | 5,375 (24.8) | 1,306 (5.3) | -0.158 | (-0.176, | -0.140) | -0.132 | (-0.1418, -0.1222) |
|  | More than 1 site | 323 (21.9) | 54 (8.9) | 1,529 (26.9) | 889 (16.9) | 0.031 | (-0.010, | 0.072) | 0.067 | (0.0396, 0.0944) |
| Geography | Metropolitan | 338 (11.2) | 62 (4.7) | 3,915 (24.7) | 922 (7.0) | -0.113 | (-0.137, | -0.089) | -0.082 | (-0.0957, -0.0683) |
|  | City | 304 (11.9) | 72 (6.1) | 2,716 (25.2) | 983 (7.3) | -0.120 | (-0.145, | -0.095) | -0.095 | (-0.1107, -0.0793) |
|  | Town | 59 (9.7) | 15 (4.9) | 273 (37.0) | 290 (8.6) | -0.236 | (-0.287, | -0.185) | -0.203 | (-0.2442, -0.1618) |
| CXR | TB signs | 564 (11.0) | 136 (5.2) | 5,463 (24.9) | 1,652 (7.1) | -0.119 | (-0.137, | -0.101) | -0.079 | (-0.0908, -0.0672) |
|  | Normal | 4 (6.9) | 4 (7.7) | 213 (21.0) | 196 (7.8) | -0.139 | (-0.259, | -0.019) | -0.264 | (-0.3071, -0.2209) |
|  | Others | 133 (13.3) | 9 (7.1) | 1,228 (27.9) | 347 (8.0) | -0.137 | (-0.206, | -0.068) | -0.156 | (-0.1834, -0.1286) |
| Bacteriological status | Confirmed | 339 (11.2) | 76 (4.4) | 3,004 (22.5) | 945 (5.4) | -0.102 | (-0.122, | -0.082) | -0.076 | (-0.0878, -0.0642) |
|  | Not confirmed | 357 (11.4) | 72 (6.9) | 3,245 (28.8) | 869 (10.3) | -0.139 | (-0.168, | -0.110) | -0.090 | (-0.1096, -0.0704) |
|  | Extra pulmonary | 5 (20.8) | 1 (16.7) | 655 (23.9) | 381 (9.2) | -0.105 | (-0.419, | 0.209) | -0.047 | (-0.0803, -0.0137) |
| Smear | Positive | 254 (11.0) | 38 (3.4) | 2,124 (24.7) | 357 (4.2) | -0.129 | (-0.154, | -0.104) | -0.104 | (-0.1197, -0.0883) |
|  | Negative | 406 (11.0) | 104 (6.4) | 2,308 (21.8) | 1,197 (7.5) | -0.097 | (-0.117, | -0.077) | -0.081 | (-0.0947, -0.0673) |
|  | Unknown | 41 (21.8) | 7 (14.0) | 2,472 (30.1) | 641 (11.4) | -0.109 | (-0.236, | 0.018) | -0.095 | (-0.1224, -0.0676) |
| Culture | Positive | 248 (10.7) | 66 (4.0) | 697 (17.5) | 676 (5.0) | -0.059 | (-0.079, | -0.039) | -0.043 | (-0.0587, -0.0273) |
|  | Negative | 249 (10.3) | 65 (6.5) | 547 (19.8) | 678 (8.0) | -0.080 | (-0.105, | -0.055) | -0.071 | (-0.0926, -0.0494) |
|  | Unknown | 204 (14.0) | 18 (11.3) | 5,660 (27.4) | 841 (10.6) | -0.140 | (-0.209, | -0.071) | -0.125 | (-0.1446, -0.1054) |

Table C. Subgroup analysis of difference in differences with propensity score matching on treatment success rate in public and private sectors in 2009 and 2014

|  | | Public | | Private | | Crude | | | Kernel (PSM) | |
| --- | --- | --- | --- | --- | --- | --- | --- | --- | --- | --- |
| Risk factors | | 2009  (n=6,195) | 2014  (n=2,803) | 2009  (n=27,396) | 2014  (n=29,988) |  |  |  |  |  |
|  |  | N (%) | N (%) | N (%) | N (%) | DID | (95% CI) | | DID | (95%CI) |
| Overall |  | 5,418 (87.5) | 2,617 (93.4) | 19,269 (70.3) | 25,148 (83.9) | 0.076 | (0.056, | 0.096) | 0.041 | (0.0292, 0.0528) |
| Gender | Male | 3,365 (86.2) | 1,652 (93.3) | 10,486 (68.3) | 13,935 (82.3) | 0.060 | (0.044, | 0.094) | 0.025 | (0.0093, 0.0407) |
|  | Female | 2,053 (89.6) | 965 (93.5) | 8,783 (73.0) | 11,213 (86.0) | 0.083 | (0.061, | 0.119) | 0.063 | (0.0473, 0.0787) |
| Age group | -19 | 868 (94.0) | 244 (96.4) | 1,166 (79.3) | 936 (94.6) | 0.121 | (0.077, | 0.179) | 0.153 | (0.1138, 0.1922) |
|  | 20-29 | 1,106 (89.6) | 491 (93.2) | 3,292 (78.3) | 3,150 (91.8) | 0.092 | (0.060, | 0.138) | 0.064 | (0.0366, 0.0914) |
|  | 30-39 | 867 (88.5) | 345 (95.6) | 3,049 (77.4) | 3,162 (93.1) | 0.077 | (0.040, | 0.130) | 0.076 | (0.0486, 0.1034) |
|  | 40-49 | 804 (84.8) | 410 (91.7) | 2,948 (71.5) | 3,791 (89.4) | 0.098 | (0.064, | 0.154) | 0.066 | (0.0386, 0.0934) |
|  | 50-59 | 636 (85.3) | 453 (92.6) | 2,692 (71.1) | 4,427 (87.7) | 0.082 | (0.045, | 0.139) | 0.116 | (0.0886, 0.1434) |
|  | 60-69 | 468 (85.1) | 280 (96.2) | 2,488 (67.8) | 3,258 (85.0) | 0.054 | (0.000, | 0.122) | 0.015 | (-0.0164, 0.0464) |
|  | 70- | 669 (82.2) | 394 (90.6) | 3,634 (58.6) | 6,424 (71.0) | 0.034 | (-0.014, | 0.096) | 0.021 | (-0.0045, 0.0465) |
| Nationality | Korean | 5,126 (87.9) | 2,412 (94.5) | 18,677 (70.9) | 24,288 (84.2) | 0.058 | (0.047, | 0.087) | 0.035 | (0.0232, 0.0468) |
|  | Others | 292 (80.7) | 205 (81.7) | 592 (56.2) | 860 (75.2) | 0.168 | (0.098, | 0.262) | 0.230 | (0.1634, 0.2966) |
| Transfer history | Treated at 1 site | 4,300 (91.1) | 2,085 (95.0) | 15,305 (70.5) | 21,289 (86.1) | 0.105 | (0.094, | 0.138) | 0.084 | (0.0722, 0.0958) |
|  | More than 1 site | 1,118 (75.9) | 532 (87.4) | 3,964 (69.7) | 3,859 (73.2) | -0.078 | (-0.124, | -0.034) | -0.092 | (-0.1214, -0.0626) |
| Geography | Metropolitan | 2,660 (87.9) | 1,242 (94.5) | 11,284 (71.2) | 11,205 (84.8) | 0.070 | (0.043, | 0.097) | 0.040 | (0.0243, 0.0557) |
|  | City | 2,234 (87.2) | 1,096 (92.6) | 7,570 (70.1) | 11,302 (84.3) | 0.088 | (0.059, | 0.117) | 0.057 | (0.0394, 0.0746) |
|  | Town | 524 (86.5) | 279 (91.5) | 415 (56.3) | 2,641 (78.5) | 0.172 | (0.107, | 0.237) | 0.128 | (0.0731, 0.1829) |
| CXR | TB signs | 4,512 (87.9) | 2,455 (93.6) | 15,565 (70.8) | 19,483 (84.1) | 0.068 | (0.056, | 0.096) | 0.033 | (0.0212, 0.0448) |
|  | Normal | 54 (93.1) | 48 (92.3) | 775 (76.4) | 2,187 (87.5) | 0.12 | (-0.018, | 0.256) | 0.242 | (0.1950, 0.2890) |
|  | Others | 852 (85.1) | 114 (89.8) | 2,929 (66.6) | 3,478 (80.3) | 0.081 | (0.010, | 0.170) | 0.120 | (0.0886, 0.1514) |
| Bacteriological status | Confirmed | 2,636 (87.03) | 1,643 (94.1) | 9,553 (71.5) | 14,660 (84.2) | 0.057 | (0.032, | 0.082) | 0.028 | (0.0123, 0.0437) |
|  | Not confirmed | 2,763 (87.94) | 970 (92.4) | 7,714 (68.3) | 6,950 (82.5) | 0.097 | (0.066, | 0.128) | 0.059 | (0.0324, 0.0716) |
|  | Extra pulmonary | 19 (79.17) | 4 (66.7) | 2,002 (73.0) | 3,538 (85.3) | 0.248 | (-0.103, | 0.599) | 0.257 | (0.2108, 0.2932) |
| Smear | Positive | 2,013 (87.03) | 1,058 (94.8) | 5,840 (67.8) | 6,943 (81.7) | 0.052 | (0.030, | 0.092) | 0.040 | (0.0204, 0.0596) |
|  | Negative | 3,264 (88.36) | 1,522 (93.0) | 7,916 (74.9) | 13,592 (85.7) | 0.056 | (0.038, | 0.086) | 0.042 | (0.0263, 0.0577) |
|  | Unknown | 141 (75.0) | 37 (74.0) | 5,513 (67.2) | 4,613 (82.0) | 0.151 | (0.022, | 0.296) | 0.152 | (0.1226, 0.1814) |
| Culture | Positive | 2,041 (87.82) | 1,557 (94.6) | 3,090 (77.6) | 11,605 (85.2) | 0.003 | (-0.016, | 0.034) | -0.0006 | (-0.0256, 0.0136) |
|  | Negative | 2,151 (88.99) | 926 (92.9) | 2,136 (77.3) | 7,200 (85.1) | 0.031 | (0.009, | 0.071) | 0.032 | (0.0065, 0.0575) |
|  | Unknown | 1,226 (84.32) | 134 (83.8) | 14,043 (68.0) | 6,343 (80.2) | 0.121 | (0.055, | 0.201) | 0.130 | (0.1084, 0.1516) |

Table D. Comparing the results of DID after using categorical and continuous variable

|  | |  | Crude (n=66,382) | | Kernel, rcs (n=66,382) | |
| --- | --- | --- | --- | --- | --- | --- |
|  | |  | DID | t | DID | t |
| with categorical | | |  |  |  |  |
|  | LTF | | -0.119 | 14.15*** | -0.087 | 17.21*** |
|  | TS | | 0.76 | 7.85*** | 0.041 | 7.20*** |
| with continuous | | |  |  |  |  |
|  | LTF | | -0.119 | 14.15*** | -0.087 | 17.16*** |
|  | TS | | 0.76 | 7.85*** | 0.041 | 7.12*** |
| *** p-value < 0.001 | | | | | | |

**[Absolute or Relative Risk]**

Table E. Absolute and relative risk differences between public and private sectors in 2009 and 2014

|  |  |  | Absolute risk difference | | | Relative risk difference | | |
| --- | --- | --- | --- | --- | --- | --- | --- | --- |
| TS | 2009(A) | 2014(B) | B-A (95% CI) |  | D-C (95% CI) | B/A*100 (95% CI) |  | F-E (95% CI) |
| Public | 87.5 | 93.4 | 5.9 (4.5, 7.5) | (C) | 7.7 (6.1, 9.5) | 106.7 (105.2, 108.4) | (E) | 12.6 (10.6, 14.8) |
| Private | 70.3 | 83.9 | 13.6 (11.5, 15.9) | (D) |  | 119.3 (116.9, 121.9) | (F) |  |
|  |  |  |  |  |  |  |  |  |
| LTFU | 2009(F) | 2014(G) | G-F (95% CI) |  | I-H (95% CI) | G/F*100 (95% CI) |  | K-J (95% CI) |
| Public | 11.3 | 5.3 | -6.0 (-4.6, -7.7) | (H) | -11.9 (-10.0, -14.1) | 46.9 (43.8, 50.0) | (J) | `-17.9 (-15.6, -20.4) |
| Private | 25.2 | 7.3 | -17.9 (-15.6, -20.4) | (I) |  | 29.0 (26.2, 31.8) | (K) |  |

TS: treatment success; LTFU: loss to follow-up; CI: confidential interval
