# Supplementary material for: Evaluating the impact of the nationwide public–private mix (PPM) program for tuberculosis under National Health Insurance in South Korea: A difference in differences analysis
Source: PLoS Med. 2021 Jul 14;18(7):e1003717. doi: 10.1371/journal.pmed.1003717 (PMC8318235; doi:10.1371/journal.pmed.1003717)
Supplement: S3 File — (DOCX) [file pmed.1003717.s003.docx]

Research Protocol^[[1]](#footnote-1)^

ver. 1.0

A Large-scale and Nationwide Public-Private Mix in South Korea: The Impact Evaluation of the Change from the Beginning in 2009 to 2014

The Korean Institute of Tuberculosis, Korean National Tuberculosis Association

Hongjo Choi

1. Background

World Health Organization (WHO) emphasized the engagement of all health care provider within national tuberculosis program (NTP) to end tuberculosis (TB)^1^. One of the approaches would be public-private mix (PPM)^2^. PPM projects implemented in the world and improved case detection rates^3^. However, due to weak enforcement of regulations and challenges in securing sustainable financing to support PPM programs, many countries have struggled in scaling up and sustaining PPM programs and many of PPM programs remain limited to pilot projects in sub-city/district areas that does not fully engage all private sector stakeholders^4^.

In this regard, the South Korean PPM program – a fully-scaled up nationwide program backed by sustainable finances from the National Health Insurance (NHI) scheme – can provide an important road map for those countries making efforts to develop plans to scale-up and sustain PPM as a nationwide program.

1. Research Purposes
2. To identify the change of treatment success rates and loss to follow-up rates between public and private sector from 2009 and 2014.
3. To identify risk factors associated with treatment success and loss to follow-up in public and private sectors.
4. Principal investigator and co-investigators

| Role | Name | Affiliation |
| --- | --- | --- |
| PI | Hongjo Choi | Korean Institute of Tuberculosis, Korean National Tuberculosis Association |
| Research associate | Sarah Yu | Korean Institute of Tuberculosis, Korean National Tuberculosis Association |
| Co-PI | Hojoon Sohn | Johns Hopkins Bloomberg School of Public Health |
|  | Hyunwoo Kim | Korean Institute of Tuberculosis, Korean National Tuberculosis Association |
|  | Kyung-Hyun Oh | Korean Institute of Tuberculosis, Korean National Tuberculosis Association |
|  | Hee-Jin Kim | Korean Institute of Tuberculosis, Korean National Tuberculosis Association |
|  | Haejoo Chung | Department of Public Health Sciences, Graduate School, Korea University |

1. Study period

One year after approval date

1. Study subject

All registered new TB cases in 2009 and 2014, using notification date from the Korean National Tuberculosis Surveillance System (KNTSS)

1. Sample size calculation and justification

We included all registered TB cases. Therefore, sample calculation is not required.

1. Recruitment

Not applicable

1. Informed consent

Not applicable. We used anonymized data from KNTSS.

1. Methodology

- We assembled cohort datasets of registered TB patients in the years 2009 and 2014, using notification data from the Korean National Tuberculosis Surveillance System (KNTSS) managed by the Korean Centers for Disease Control and Prevention (KCDC)Measurements.
- We included only the newly identified (coded in the KNTSS as ‘new cases’) TB patients who started their TB treatment in the respective cohort years who were not previously registered in the system in years before the designated cohort years (notified in 2001 ~ 2008 for 2009 cohort and 2001 ~ 2013 for 2014 cohort).
- The private sectors included university hospitals, private secondary and tertiary hospitals, and primary private clinics, while the public sector included public health centers and national hospitals
- Comparison of the baseline covariates – age, gender, nationality, transfer history (treatment reported at one or more institutions since reported as a TB case), geographic division (categorized as metropolitan, city, and towns designated), diagnostic test results (chest X-ray, smear, and culture) – were assessed using the Chi-square test.
- To assess the causal effect of the Korean PPM strategy on TB treatment outcomes, we used the difference-in-difference (DID) analysis with an assumption that, in absence of the policy intervention (or in this case, early policy intervention), unobserved differences between the compared groups would be the same over the 5-year period. In our analysis, we examined the differences in the differences of outcome variables between public and private sectors that changed over time.
- To address the robustness of our main DID analysis estimate, we performed propensity score matching (PSM) analysis to match individuals on key variables (age, gender, nationality, transfer history, diagnostic test results) in the public sector with similar individuals in the private sector.
- Data curation and analyses were performed using Stata v15.0 (StataCorp. College Station, TX, USA)

1. Safety evaluation

Not applicable

1. Anticipated side effects and cautions

Not applicable

1. Criteria for withdrawal

Not applicable

1. Risk and Benefit for study participants

Not applicable

1. Privacy protection
2. All data was anonymized and provided from KCDC. No identification numbers were provided to investigators.
3. We store all data for three years after the study completion according to the Bioethics and Safety Act.
4. References

1. World Health Organization. The Stop TB Strategy: Building on and enhancing DOTS to meet the TB-related Millenium Development Goals. Geneva: WHO/HTM/TB; 2006.

2. Murthy KJR, Frieden TR, Yazdani A, Hreshikesh P. Public-private partnership in tuberculosis control: experience in Hyderabad, India. *The International Journal of Tuberculosis and Lung Disease* 2001; **5**(4): 354-9.

3. Lei X, Liu Q, Escobar E, et al. Public-private mix for tuberculosis care and control: a systematic review. *International Journal of Infectious Diseases* 2015; **34**: 20-32.

4. Baloch NA, Pai M. Tuberculosis control: business models for the private sector. *Lancet Infectious Diseases* 2012; **12**(8): 579-80.

1. The protocol submitted and approved by Korean National Tuberculosis Association Institutional Review Board. The protocol originally was written in Korean and this version was translated and summarized by authors. [↑](#footnote-ref-1)
