# Supplementary material for: Evaluating the impact of the nationwide public–private mix (PPM) program for tuberculosis under National Health Insurance in South Korea: A difference in differences analysis
Source: PLoS Med. 2021 Jul 14;18(7):e1003717. doi: 10.1371/journal.pmed.1003717 (PMC8318235; doi:10.1371/journal.pmed.1003717)

| TB Case Notification Form (2014 version) | |
| --- | --- |
|  |  |
| To: Director of ___________ Health Center | Fax: __________________ |
| Incidence Report (#1~#32) | |
| **[Demographic characteristics of patient]** | |
| 1. Name: | 1. ID number: |
| 1. Age: | 1. Gender: [ ] M, [ ] F |
| 1. Nationality (Only foreigner): | 1. Entry date (Only foreigner): Y M D |
| 1. Telephone number: | 1. Mobile phone: |
| 1. Address and Zip code: | |
| (10) Occupation: [ ] Teacher [ ] Health care worker [ ] hair dresser [ ] Grocery service worker [ ] Sailor [ ] Aircrew [ ] Student [ ] Others (______) | |
| (11) Workplace (school) address: | Name of workplace (school) |
| **[Previous TB history]** | |
| (12) Previous TB history: [ ] Yes, [ ] No | (13) Duration of previous TB treatment: [ ] less than 1 month, [ ] 1 month or more |
| (14) Number of previous TB treatment episodes: [ ] one, [ ] two or more | |
| [BCG history] ※ Only less than 15 years old | |
| (15) BCG scar: [ ] Yes, [ ] No | (16) Methods of BCG: [ ] Intradermal, [ ] Transdermal. [ ]Unknown |
| **[TB initial diagnosis]** ※ Check if available | |
| (17) Sputum smear: [ ]Positive, [ ]Negative, [ ]Unknown, [ ]Not done | (18) Sputum culture: [ ]Positive, [ ]Negative, [ ]Unknown, [ ]Not done |
| (19) Smear (non-sputum): [ ]Positive, [ ]Negative, [ ]Unknown, [ ]Not done | (20) Culture (non-sputum): [ ]Positive, [ ]Negative, [ ]Unknown, [ ]Not done |
| (21) Biopsy: [ ]Positive, [ ]Negative, [ ]Unknown, [ ]Not done | (22) Other (eg. PCF): [ ]Positive, [ ]Negative, [ ]Unknown, [ ]Not done |
| (23) Radiology: [ ]Normal, [ ]TB suspicious, [ ]Unknown, [ ]Not done | (24) PPD test: Induration size( mm), [ ]Not done |
| (25) IGRA test: [ ] Quantiferon In-Tube( IU/ml), [ ] Quantiferon TB-Gold( IU/ml), [ ] T-SPOT(number: ), [ ] Not done | |
| **[ICD-10 code]** | |
| (26) Code #1: _ _ _ . _ | (27) Code #2: [ ]U88.0, [ ]U88.1 |
| **[Treatment details]** | |
| (28) Initial visit date: Y M D | (29) Follow up management: [ ] Yes (start date: Y M D), [ ] No |
| (30) Category: [ ]New case, [ ]Previously treated case, [ ]register after failure,. [ ]register after loss to follow up, [ ]transfer in, [ ]chronic case, [ ]Others | |
| (31) Treatment initiation date or diagnosis date: Y M D | |
| (32) Regimen: [ ]H, [ ]R, [ ]E, [ ]Z, [ ]Rfb, [ ]Km, [ ]Amk, [ ]S, [ ]Lfx, [ ]Mfx, [ ]Ofx, [ ]Pto, [ ]Cs, [ ]PAS, [ ]Lzd, [ ]Clr, [ ]Others( ) | |
|  | |
| Treatment Outcome Report (#33 and 34) | |
|  | |
| **[Treatment outcome]** | |
| (33) Treatment termination date: Y M D | |
| (34) Treatment outcome: [ ] cure, [ ] completion, [ ] failure, [ ] loss to follow up, [ ] transfer out, [ ]died ([ ]TB-related death, [ ]other caused death), [ ]diagnosis change ([ ]NTM, [ ]Cancer, [ ]others), [ ]Others | |
| I am reporting the tuberculosis case above according to the TB prevention act | |
| **[Treatment institution]** | |
| Date of report: Y M D | |
| ID of the institution: | |
| Name of the institution: | |
| Address: | |
| Name of physician: (signature) | |


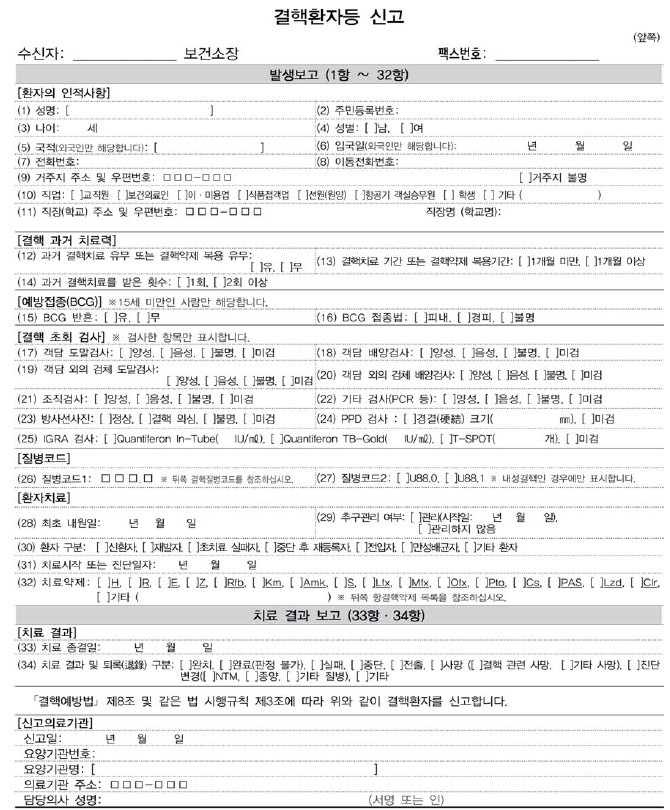

Supplement: S4 File — TB, tuberculosis. (DOCX) [file pmed.1003717.s004.docx]
