# Supplementary material for: Evaluating the impact of the nationwide public–private mix (PPM) program for tuberculosis under National Health Insurance in South Korea: A difference in differences analysis
Source: PLoS Med. 2021 Jul 14;18(7):e1003717. doi: 10.1371/journal.pmed.1003717 (PMC8318235; doi:10.1371/journal.pmed.1003717)
Supplement: Attachment — Submitted filename: yu.pdf [file pmed.1003717.s005.pdf]

This paper reports a before and after study of cohorts of tuberculosis patients in South Korea.

I was asked for a statistical report and I interpret that to include all aspects of the design and conduct of the study.

Points of detail

Page 3 I did not understand the sentence starting 'In 2017' as it seemed to contradict itself.

Page 5 I think I might give a date as international readers might not all know when the Korean War took place.

Pages 6 and 7 I think we might need a justification of why these exclusions were needed. Some of these are perhaps more for the clinical reader but I cannot see any statistical reason presented here which might justify removing people with missing covariate information.

Page 7 Age is an inherently continuous variable and categorising such a variable wastes information (Altman and Royston, 2006; Royston et al., 2006) and leads to models which are often implausible as they predict the effect remaining flat within categories and then jumping to a new value at the category boundary.

Page 9 I think it might be good to state explicitly that the authors' models yield absolute risk differences for the benefit of readers for whom this is not going to be immediately apparent.

Page 9 The description of the β values is not complete and rather confusing. Ignoring β_0 which is scientifically uninteresting we need a description of each of the other three. More importantly we would benefit from a table in the results which gives us the estimated values for these with their confidence intervals. At the moment this is scattered through the text and figures.

Page 11 If the sub group analyses are important then they need presenting fully rather than summarised. Since the authors do mention them in the discussion I assume they are important.

Page 24, Figure 5 What is the message behind this figure? I cannot see why the reader should have to scan the whole figure to find all the age results and similarly for region and the other predictors. Would it not be better to group all the levels of a predictor together? Is there some

reason why TSR01 is in red? Presenting the predictors in a different order in the two sub–graphs also hinders interpretability.

Points of more substance

Propensity score matching

This is presented in the methods but we do not see much detail about its success. Was this done to yield one to one matches and if so how many patients remained unmatched? If it was done to generate strata then how were they used in analysis and was the distribution of the two groups similar?

Which is the primary analysis?

On page 9 the authors state that their propensity score matched analysis was a sensitivity analysis but in the results section it seems to have become the primary analysis. Why is this?

Absolute or relative risk

The authors have chosen to model absolute risk by using proportions on a linear scale. This is fine and in many ways superior to relative risks on the logit or log scale but I wonder whether given the closeness of some of the proportions to unity or zero we need to have the results supplemented by relative values. For example if we look at Figure 4 the private sector started at 0.252 and fell to 0.073 a crude drop of 0.179. Since the public sector started at 0.113 it cannot possibly have achieved the same absolute risk reduction. Similar issues arise elsewhere.

The parallel trends assumption

The difference in differences model relies on the parallel trends assumption. Is there any evidence that any changes in the period before 2014 were at the same rate in both public and private sectors? If not then the authors can presumably access earlier years to check and re–assure us.

Summary

Points for clarification and some suggestions for enhancement of the analysis.

Michael Dewey

References

D G Altman and P Royston. The cost of dichotomising continuous variables. *British Medical Journal*, 332:1080, 2006.

P Royston, D G Altman, and W Sauerbrei. Dichotomizing continuous predictors in multiple regression: a bad idea. *Statistics in Medicine*, 25:127–141, 2006.
