# Supplementary material for: Evaluating the impact of the nationwide public–private mix (PPM) program for tuberculosis under National Health Insurance in South Korea: A difference in differences analysis
Source: PLoS Med. 2021 Jul 14;18(7):e1003717. doi: 10.1371/journal.pmed.1003717 (PMC8318235; doi:10.1371/journal.pmed.1003717)
Supplement: Attachment — Submitted filename: Peer review Choi et al.docx [file pmed.1003717.s006.docx]

The publication by Choi et al. presents an evaluation of the public-private partnership model in South Korea and is interesting for several reasons:

i) the model is unique in that it capitalises on the universal health coverage
ii) South Korea has seen a rapid decline of TB incidence (a situation other countries will hopefully face in the next decade) – however compared to other high resource settings, TB incidence is still relatively high
iii) the analysis is innovative using a difference in difference methods and propensity score

Different models of public-private partnership for TB control in different setting rightly deserve focused attention and would be well suited to be published in Plos Medicine.

As a general note, the manuscript would benefit from editing by a native (scientific) English speaker. I am myself not a native speaker, thus I have limited my comments on grammar and language to those paragraphs which could be considerably shortened or need clarifications.

Please find my detailed comments below:

*Introduction*

Line 71: National Health Insurance Scheme – this needs to be explained in more detail, otherwise the reader will not be able to follow the discussion.

Line 74-76: This sentence is extremely difficult to understand especially without a background of how the Korean TB program looks like.

*Methods*

General comment:

In order to understand the PPM and put it into context - it would be important to describe the South Korean TB programme. Where are patients diagnosed – in hospitals or outpatients? Are there dedicated TB clinics (i.e. is it a vertical program) or are TB patients seen in chest clinics. How is treatment managed programmatically – are most patients initiated in a hospital or as outpatients. If patients are initiate in hospital, how long do they stay in hospital and where do they received their outpatient care (at their GP, in an outpatient TB clinic affiliated to the hospital?

From a methodological point of view my main question is – why did the authors decide to use 2 cohorts rather than looking at the trends longitudinally – i.e. 2009-2014 spanning the period pre-implementation, scale-up and post implementation.

Paragraph: “Public private mix in South Korea”

I would recommend to restructure this sexction, as it is difficult to follow. For example the second paragraphs describes the 2007 pilot and the 2011 nationwide roll-out of PPM, while the third paragraph mentions a much earlier pilot in 2001. The authors may want to include a figure describing timeline both with regards to the national health insurance scheme and the PPM

Line 92: consider replacing “rapid growth in TB patient” with maybe “more TB patients seeing care..”

Line 100: who pays the PPM nurses?

Line 108-109: “Modeled based on..” please edit – this might be “Models based on

Line 121-123: “Private or public sector designation for each TB patient in the respective cohort years was assessed based the primary institution reporting registration of patient’s TB.” Please edit for example “Each TB patients was categorised as receiving private or public sector care based on the designation of the institution registering the TB patient in with the NTP”

Line 125-126: “We included only the newly identified (coded in the KNTSS as ‘new cases’) TB patients who started their TB treatment in the respective cohort years who were not previously registered in the system in years before the designated cohort years (notified in 2001 ~ 2008 for 2009 cohort and 2001 ~ 2013 for 2014 cohort). Please edit “…TB patients who started TB treatment and were not previously registered for TB (since 2001)”

Line 130-131: “We excluded patients with inappropriate treatment period (cases that completed treatment before treatment initiating date)…” I am not sure I understand this part of the sentence. Does that mean a patient was registered and their date of completing treatment was incorrectly entered as being before treatment initiation?

Line 143: – please include “variable” after binary.

Line 149-152: “Patient’s examination for the TB diagnosis was classified based on three types of diagnostic test results. Chest X-ray (CXR) results were classified as those with “presumptive TB”, “normal”, or “other disease”. Bacteriological status was classified based on the ICD-10 code recorded in the KNTSS database (A15.X:

152 bacteriologically confirmed, A16.X: not confirmed, others: extrapulmonary).”

What was the third diagnostic test? Or do you mean each diagnostic test had three possible result categories? I think the wording of “presumptive TB” is not the best for a CXR – I would categorise a CXR as “findings suggestive of TB”. What was the means of bacteriological confirmation – culture, smear, molecular tests? Extrapulmonary TB can also be microbiologically confirmed – would these cases be classified as bacteriologically confirmed or others?

Line 153-155: “Data on LTFU and treatment success (TS) were assessed as outcomes where the results of treatment were classified as cure, completion, failure, death, or LTFU in the KNTSS database” Please rephrase for example “treatment success was categorized as cure, completion, failure, death, or LTFU as per the KNTSS database”.

Line 155-158: “The LTFU rate was defined as the sum of patients with unknown treatment results (marked in the database as ‘not evaluated’) and interrupted treatment (marked as ‘interrupted’) over a total number of new TB patients registered on KNTSS within each designated cohort year.” Please consider editing, for examples “Patients were defined as LTFU if they had unknown treatment outcomes or interrupted treatment.” Generally a rate has an time element to it (usually the denominator is person-years). What the sentence is describing is a proportion – i.e. the number of patients LTFU over the total number starting treatment

Line 159-161: “Those patients who were transferred to another institution (public or private) with more than months of treatment data missing post transfer were also marked as LTFU.” – I am not sure I understand why this is mentioned separately. In a previous sentence it states that LTFU also include those with treatment interruption (it does not mention how long somebody who have to interrupt treatment to be classified as treatment interruption).

Line 169-172 “To assess the causal effect of the Korean PPM strategy on TB treatment outcomes, we used the difference-in-difference (DID) analysis with an assumption that, in absence of the policy intervention (or in this case, early policy intervention), unobserved differences between the compared groups would be the same over the 5-year period.” My understanding of the study design was that the authors created one cohort of patients registered in 2009 and followed them up until treatment completion and one cohort registered in 2014 with follow-up until treatment completion. The study does not span a period – it provides two different snapshots.

Line 186-188 “PSM was done to address potential biases that may be associated with the confounding factors arising from imbalances in the observed covariates from our non-longitudinal and non-counterfactual nature of our comparator groups.” Please rephrase “PSM was performed to address potential biases that may be associated with the confounding factors arising from imbalances in the observed covariate because of the non-longitudinal and non-counterfactural nature of the analysis.”

*Results*

Line 200-201 “patients managed in the public sector decreased (6,195 in 2009 → 2,803 in 2014)” please replace by (n=6195 (%) in 2009 and n=2803 (%) in 2014). Please consider doing this throughout the result section.

Line 212: please include p value

Line 214-216: is this truly rate and not proportion?

Line 219: “with both sectors” needs to be replace by “in both sectors”

Line 226: “higher age groups” please consider to replace by “older patients” or “in the older age-group”

Line 229-232 “Particularly, patients with transfer history were negatively impacted by the PPM program with 9.2% (95% CI, 6.3 to 12.1%) reduction in TSR and 6.7% (95% CI, 4.0 to 9.4%) increased LTFU rates in the private sector compared to the public sector patients in this subgroup over the 5-year period.” Maybe consider splitting this sentence in two to make it clearer.

Lines 237- 241: “In crude DID analyses, certain patient sub-groups (patients with normal CXR results and EP-TB for TSR; and patients with EP-TB and unknown smear status for LTFU) had contradicting interpretation to that of PSM adjusted DID estimates which indicated that patients in these subgroups did not haves significant improvements in their treatment outcome as a result of the PPM.” Please edit.

*Discussion*

Line 253*:* replace “in” with “which”

Line 262-266: “Furthermore, alongside of PPM program expansion, continued expansion of NHI on TB service coverage and alignment of the comprehensive National Strategic Plan for TB control with relevant updates in government regulations and mandates have effectively lowered both financial and health systems barriers for patients suffering from TB-illness.” Please consider rephrasing.

Line 266: replace the word “eliminate”

Line 276: what is meant with policy blind spot?

Line 289-290: “This is because repeated measure of same population is not feasible from active TB cases.” The authors could have used the population as a whole as a denominator. However, I think the problem is that one does not know what the denominator of the population is who access private/public care is. The authors may want to rephrase the sentence.

Figure 1: What does eligibility 100 TB notifications in a year mean? Explain either in the legend or in the figure.

Figure 3: I do not understand the purpose of this figure – a bit more explanation may be needed (i.e. a legend)?

Figure 5: The reasons why the TSRU needs to be explained. There is no x-axis. Rather than using abbreviations I would write the meaning of the categories at the left side of the figure.
