# Supplementary material for: Evaluating the impact of the nationwide public–private mix (PPM) program for tuberculosis under National Health Insurance in South Korea: A difference in differences analysis
Source: PLoS Med. 2021 Jul 14;18(7):e1003717. doi: 10.1371/journal.pmed.1003717 (PMC8318235; doi:10.1371/journal.pmed.1003717)
Supplement: Attachment — Submitted filename: PMEDICINE-D-19-03769R2_rebuttal_final2.docx [file pmed.1003717.s007.docx]

Requests from the editors:

Please briefly explain the reasons that full access cannot be provided to study data, in your data statement.

Please also add the URL for KCDC, or a point of contact via email.

🡺 Thank you for this comment. We’ve added following statement for ‘data availability’:

“Data cannot be shared publicly because of the data sharing policies at the Korea Centers for Disease Control and Prevention Agency (KCDA), governed by the Statistics Act Enforcement Ordinance Article 17 Item 2 (“Procedures and Methods for Statistics-based Policy Evaluation” available at: <http://kostat.go.kr/portal/eng/aboutUs/5/6/index.static>). Data are available only for researchers who meet the criteria for access to confidential data, subject to approval from respective ethics review committee at the KCDA.”

Please restructure your title by moving the country name before the colon (e.g., "... in South Korea: a difference ...").

🡺Thank you for this suggestion. We’ve revised the title accordingly as the following:

“Evaluating the Impact of the Nationwide Public-Private Mix (PPM) Program for Tuberculosis under the National Health Insurance in South Korea: A Difference in Difference Analysis”

In your abstract, please remove "highly" from "highly privatized".

🡺 We’ve removed the word “highly” from the specified text in the abstract (see line 57)

Please quote aggregate demographic details for study participants in your abstract.

🡺 Thank you for this suggestion. We have added the aggregate demographic details for study participants in the abstract (see line 66-69).

“In both the private and public sector, the proportion of patients with transfer history decreased (public: 23.8% to 21.7% and private: private 20.8% to 17.6%) and bacteriological confirmed disease increased (public: 48.9% to 62.3% and private: 48.8% to 58.1%) in 2014 compared to 2009.”

Please trim your author summary. The three individual subsections should each consist of 3-4 points, each consisting of no more than 1-2 short sentences each.

🡺 We have revised the author summary accordingly.

Please ensure that you refer to the attached STROBE checklist in the Methods section ("See S1_STROBE_Checklist" or similar).

🡺 We referred to the attached STROBE checklist in the Methods section (line 332-333):

“This study is reported according to the Strengthening the Reporting of Observational Studies in Epidemiology (STROBE) guideline (S1 STROBE Checklist).”

At line 381 and any other instances, please add "to our knowledge" if you wish to claim "the first", for example.

🡺 Thank you for this feedback. We have revised the text throughout our manuscript accordingly (lines 102, 122, & 391)

Please quote exact p values or "p<0.001" throughout the paper, unless there are specific statistical reasons to quote smaller p values.

🡺We have replaced “p<0.0001” with “p<0.001” throughout the manuscript (Lines 71, 72, 359, 369, 379, 380). For other p-values, we have quoted exact values.

Please substitute "sex" for "gender" throughout the text, where appropriate.

🡺(SR) We have replaced “gender” with “sex” throughout the manuscript. (lines 256, 260, 297, 312, 320, & Table 1)

We suggest "difference in differences", throughout.

🡺(SR) We have substituted “difference in differences” for “DID” accordingly throughout the manuscript.

Please remove the information on study funding and competing interests from the end of the main text. In the event of publication, this information will appear in the article metadata via entries in the submission form.

🡺Thank you for this comment. We have removed the information on study funding and competing interests in the main text and updated relevant information in the manuscript submission form.

Please revisit your reference list to ensure that all citations meet journal style. For example, 6 author names should be listed rather than 3, followed where appropriate by "et al.". All italics and boldface text should be converted to plain text.

Please abbreviate journal names as appropriate, and, noting reference 30, ensure that all citations include full access information.

🡺For above two comments, we have reviewed all of our reference citation styles and updated them according to the journal requirement/style (e.g. listed up to 6 author names and formatted all texts to plain text, and included full access information for all citations included in our references).

Please adapt the attached STROBE checklist so that individual items are referred to by section (e.g., "Methods") and paragraph number rather than by page or line numbers, as the latter generally change in the event of publication.

🡺 Thank you for this notion. We’ve revised STORBE checklist to refer relevant ‘sections’ of the manuscript and paragraph number rather than by page/line numbers.

Reviewer 1 comments

The authors have addressed all my points except for one small matter.

They have now clarified the coefficents but I also suggested these could be presented together rather than scattered through the manuscript. I still think this is a good idea but I cannot seem to find it. Have I missed something?

🡺Thank you for your comment on this matter. Contrast to your notion, we believe we’ve presented the coefficients for the difference in difference analysis in a single paragraph (i.e. not scattered throughout the manuscript) in lines 321-327:

“The estimated coefficients have the following interpretation. $\hat{\beta_{0}}$ is the mean outcome for the public sector (control) in 2009. $\hat{\beta_{0}}+\hat{\beta_{1}}$ is the mean outcome for the public sector (control) in 2014. $\hat{\beta_{2}}$ is the single difference between private (intervention) and public (control) groups in 2009. $\hat{\beta_{3}}$ is the DID estimate quantifying absolute differences in TB treatment outcomes between 2009 and 2014 in patients managed in the private sector compared to those in the public sector as a result of the nationwide PPM intervention.”

If this is not what you are referring to, we would very much appreciate it if you could specify a couple of references (i.e. scattered descriptions/presentation of the coefficients) with line references. Thank you!

Reviewer 4 comments

Review of " Impact Evaluation of Nationwide Public-Private Mix (PPM) Program for Tuberculosis under National Health Insurance: A Difference-In-Difference
Analysis of the South Korean PPM"

This paper provides an interesting analysis on an important topic. I only have a few minor comments on the paper:

1.    The authors refer to the WHO goals on Tuberculosis. They might consider mentioning Sustainable Development Goal #4 (which includes sub-goals for TB as well) as part of the motivation.

🡺 Thank you for this suggestion. We’ve revised first couple of sentences in the introduction to include ‘Sustainable Development Goal for TB accordingly. Please see lines 135 - 139:

“The global Tuberculosis (TB) control efforts are faced with the perpetual problems of the ‘missing millions’ of TB patients – one-third of estimated TB cases – who are left undiagnosed or without treatment. [1-3] These consequences of TB care cascade gaps are major obstacles in achieving the ambitious post-2015 global TB goals and Sustainable Development Goals Target 3.3 [4-7] and are most prominent in the private sector.”

2. The authors mention that PPM-dedicated nurses provide patient education and treatment adherence. What is included in the patient education? While this may not be so important for the statistical analysis of treatment success (or is it?), it would be useful to understand what the program does in this respect.”

🡺 Thank you for your comment and suggestion. We’ve included additional text in our methods section to provide additional description on patient management and education in the South Korean Public-Private Mix program. The revised text is as follows (Line 224-231):

“1) registering TB patients and regularly updating patient information in the KNTSS; 2) educating TB patients (contents include: informing patients on the importance of TB treatment and adherence to the treatment schedule, risks of development of drug resistance, basic knowledge of TB disease, a need for contact investigation for household members and frequent contacts of the patients, and potential treatment side effects and how to cope/manage these side effects); 3) phone-call reminders prior to patient’s treatment follow-up appointments; 4) checking patient’s TB treatment adherence and anti-TB drug adverse events; 5) follow-up calls for those patients missing their clinic visit appointments.[21].”

3.    The paper mentions that patients with drug-resistant TB were removed from the sample. How many are there of these in the data? Also, it would be useful to know how big of a problem drug resistant TB is in South Korea.

🡺 Thank you for your comment. In our original data, 0.3% and 1.6% of all patients in the KNTSS database used for our study for 2009 and 2014 cohorts were DR-TB patients (see Figure 2). We’ve revised our manuscript text to include actual number and percentage of DR-TB patients in the original KNTSS data used for our study (lines 252-254). Also, in Figure 2 (Flow diagram of our study population inclusion and number of patient data excluded with specified reasons) we also provide the same information regarding DR-TB patient data.

In South Korea, the proportion of DR-TB patients reported amongst all TB cases averaged around 2% annually for the past 10 years (KCDA. (2019). Annual Report on the Notified Tuberculosis in Korea. Osong: Korea Disease Control and Prevention Agency.

Revised text in the manuscript is show below (lines 252-254):

“drug-resistant (DR) TB (96 and 538 patients in 2009 and 2014 cohorts: represents 0.3% and 1.6% of all patients in the original KNTSS data used for this study)”

4.    There is a literature in economics which analyses the impact of historical TB policies using differences-in-difference type techniques (e.g. Anderson et al., (American Economic Journal: Applied Economics, 2018); Egedesø et al. (Economic Journal, 2020), Clay et al. (Journal of Development Economics, 2020). Given the focus on TB and the overlap in technique, I think these papers deserve to be cited in the paper.

🡺 Thank you for these very interesting articles. We have revised the text in our manuscript to refer to these articles in the context of difference in difference techniques used to evaluate TB policies/campaigns (lines 302 - 304):

“In the field of TB, difference in difference analysis have been previously used to evaluate long-term effects of pre-1950s TB policies/public health campaigns (limited to the US and Denmark) using historical data [29-31].”

5.    Table 1 gives information on how the treated and the control group differ in terms of observable characteristics. The table looks somewhat messy. I would suggest stating the total subsample for the treated and non-treated and then show everything else in percentage terms, e.g. the percentage of men.

🡺 Thank you for your suggestion. We have revised our Table 1 accordingly. In doing so, we removed columns of showing comparisons between 2009 and 2014 cohorts. Instead, we add additional footnotes for parameters that were statistically significant in differences of proportion between 2009 and 2014 cohorts. We hope these revisions adequately reflect your suggestions.

6.    S2 Figure A provides support for the parallel trend assumption. From 1993 to 2009, the trends look approximately parallel. Yet, from 1987 to 1993, something seems to be going on in the private sector. Is there any policy that could explain this? Parallel trends seem a reasonable assumption given the long period in the 1990s and 2000s in which the development looks parallel, so I do not think that is a big problem, but it would be useful to make some comment on this.

🡺 Thank you for your comment. As of 1989, the National Health Insurance (we have provided brief description of the NHI system and its history in our manuscript text: lines 408-411) formally covered the entire South Korean population. Hence, it is conceivable that the trends seen between 1987 and 1993 (Figure S2) is likely attributable to the improvements in NHI coverage for health services provided in the private sector. We have provided additional text on this matter in the manuscript in lines 407 - 409:

“the South Korean NHI scheme reached full national coverage by 1989 and improved coverages for health services provided in the private sector [16].”

Reviewer 5 comments

Review of Yu et al
M/s: Impact Evaluation of Nationwide Public-1 Private Mix (PPM) Program for Tuberculosis under National Health Insurance: A Difference-In-Difference Analysis of the South Korean PPM

Note: Although this is a revised manuscript, and includes responses to previous reviewers, this is the first time that the current reviewer has seen this manuscript.

Summary
This manuscript used a difference-in-difference (DID) analysis and compared the changes in treatment outcome (treatment success (TS) and loss to followup (LTFU)) between the public and private sector TB patient cohorts in 2009 and 2014. The baseline covariates examined were: age, gender, nationality, transfer history (treatment reported at one or more institutions since reported as a TB case), geographic division (categorized as metropolitan, city, and towns designated), and diagnostic test results (chest X-ray, smear, and culture). The primary result being claimed is that the PPM intervention applied only to the private sector patients resulted in a faster improvement trend/achievement for the private sector patients vs public sector patients.

Overall assessment
On the positive side, this is an important topic in public health (how to improve quality in private sector provision), the Korean approach is impressive and critical to document, and it is good to see a more rigorous than usual analysis of programmatic TB data.
On the negative side, the result may not be greatly surprising, given the treatment success data available from KCDC at the start of the study, and there are some questions around how the results are analyzed and presented (see below for the 2 major comments - which the authors may well be able to respond to successfully).

Major comments
1) There is a lack of clarity in describing the main results.
Page 59-60, line 350: "Although TS improved in both sectors by 2014 (to 93.4% and 83.9% respectively in 2014), improvements in the private sector (Figure 4 A & B, red lines) was at least 4.1% (p value<0.0001 with 95% Confidence Interval [CI], 2.9 to 5.3%) higher than that of the public sector (Figure 4 A & B, blue lines)."
First off, this is referring to Fig 3, not Fig 4.

🡺 Thank you for picking up on this error. We’ve corrected this error while address your comments below.

Second, it is really difficult for the reader to understand: (a) where the 4.1% in the text comes from (what is it referring to; how do you derive that from Fig 3); and (b) what we are actually meant to conclude from Fig 3. Most readers from the TB side (including this one) are not used to interpreting DID scores, or at least need them to be more clearly explained, including an explanation of which parts of Fig 3 we are meant to be comparing. Same set of issues for the 8.7% in the next paragraph related to LTFU. I would suggest you need something really simple such as: "The total percentage of increase in TS between 2009 and 2014 (red lines, Fig 3B) was greater for private sector patients (0.839-0.703=0.136) than for public sector patients (0.93-.836=0.094), leading to a positive DID of 4.2% (0.136-0.094=0.042, or 4.2%)." This is not interpretation of the results (which would belong in the discussion) but explanation of how to read Fig 3 (which belongs properly in the Results section, which is what I'm referring to here). The same kind of fix is needed for the following paragraph about LTFU.

🡺 Thank you for pointing to this issue. To clarify how “4.1%” was derived from our analysis, we revised the texts between lines 354 and 362 as the following:

“During the first five years of the scale-up of the of formal nationwide PPM program, TS improved from 87.5% to 93.4% (absolute improvement of 9.10%) and 70.3% to 83.9% (absolute improvement of 13.3%) in the public and private sector respectively (Table 2). Although TS was consistently lower in the private sector, a positive difference in difference (DID) estimate of 0.041 (or 4.1%; 95% Confidence Interval [CI], 2.9 to 5.3%) – calculated based on the differences of the absolute improvements in TS rate in the respective sector (13.3% - 9.10%) – infers that the rate of improvement was higher (or impact of the scale-up of the nationwide PPM was greater) in the private sector (Figure 3B, red lines) relative to the public sector.”

Likewise, we have revised the texts for LTFU in lines 363 - 371 accordingly:

“For LTFU, the private sector had significantly higher proportion of LTFU (25.2%) in the baseline year relative to the public sector (11.3%) in the inception year of the nationwide PPM (Table 2). In 2014, the proportion of TB patients LTFU out of all patients enrolled in TB treatment fell to 7.3% and 5.3% in the private and public sector respectively, achieving an absolute decrease in LTFU of 2.0% and 10.7% for the respective sectors. A negative difference in difference (DID) estimate of -0.087 (or -8.7%; 95% Confidence Interval [CI], -9.7% to -7.7%, p-value < 0.001) – calculated based on the differences of the absolute decrease in LTFU (-2.0% - 10.7%) – indicates the rate of decline in LTFU was faster in the private sector (Figure 3B, blue lines) compared to the public sector as a result of the PPM expansion.”

2) My biggest question: why is DID the right and most fair way to make this comparison? I've leave the final determination about statistical methods to reviewers 1 and 3, who seem much more knowledgeable on this. But I have a point based on logic that I hope those reviewers can also assess.
That point goes as follows: What I care about most as a TB person is the rate at which unfavorable treatment outcomes are being replaced by favorable treatment outcomes. As noted in the first paragraph of the discussion, the "gaps in TS and LTFU" are what we really care about. So, what is the rate of improvement in these gaps?
Therefore, instead of DID, I can use Fig 3B to look at the proportion of unfavorable LTFU outcomes that are being eliminated between 2009 and 2014. By 2014, public sector manages to get rid of (0.145-0.053)/0.145=63% of their original, 2009 proportion of unfavorable LTFU outcomes. In the same period, private sector gets rid of (0.252-0.073)/0.252=71% of their original unfavorable outcomes. So private sector does indeed get rid of a higher proportion of the poor outcomes that they started with in the 2009 cohort. To me, that would be a clearer and more intuitive way to present these same results.

For TS, we can do a similar analysis. But here we ask what proportion of ALL unfavorable outcomes (1-TS, which includes LTFU and other poor outcomes such as death) were eliminated over time.  This calculation for public sector is: ((1-.836)-(1-.93))/(1-.836)=(0.164-0.07)/0.164= 57% of all unfavorable outcomes from 2009 were eliminated by the time we got to 2014.
And the same calculation for the private sector: ((1-.703)-(1-.839))/(1-.703)= (.297-.161)/.297= 46%.
So actually in terms of getting rid of all unfavorable outcomes, the proportion of improvement was worse for the private sector than it was for the public sector (even though the absolute gain was greater for private, since its original deficit was so much bigger). If my calculations are correct, this somewhat undermines the central message of the paper, and at least needs to be acknowledged.

🡺 We agree with the author’s assessment that we are ultimately interested in assessing how “unfavorable treatment outcomes are being replaced by favorable outcomes” as a result of the PPM intervention (and its scale-up during the first five years as a formal nationwide program). We also agree with the general analysis ‘concept’ presented by the reviewer, which for the LTFU is consistent with the difference in difference analysis. However, for 1-TS analysis, we need to be a bit careful when combining two very different policy level impact of the PPM intervention on the patient important outcomes in a single estimate such as the one presented by the reviewer.

From our perspective, it was important to consider the limits of policy impact of the PPM intervention. Likewise, we feel that assessing policy level impact of the PPM intervention on improving treatment interruption (e.g. loss-to-follow-up, treatment success) and deaths are very different in nature. This is largely due to two main issues. First, the main objective and programmatic components of PPM intervention focuses on improving patient adherence to TB treatment. For this, indicators such as treatment success, loss to follow-up and treatment failure are more appropriate patient-important outcomes to assess the policy impact of the PPM intervention. Second, when considering death as an outcome to evaluate impact of the PPM intervention, we have to consider potential attributes of death more closely: patient’s age (TB disease more concentrated in the elderly population can lead to more observation of death regardless of the PPM intervention) and the degree of more serious illness managed in the respective sector. In South Korea, 1) TB disease burden is higher in elderly population and 2) private sector (which includes general or teaching hospitals and/or tertiary hospitals) generally manages more serious cases of TB (for this, data is not available to evaluate this effect in our analysis).

As such, in Table 2, we can see that 1) both sectors saw an increase in death between 2009 and 2014 (public: 0.73% to 1.25% vs. private: 4.35% to 8.75%) and 2) the private sector saw much higher percentage of deaths than those in the public sector public. Applying the same calculation concept by the reviewer to ‘death’, we find that the rate of increase was higher in the private sector ((8.75-4.35)/4.35 = 102% of the original estimate from 2009) vs. that of the public sector ((1.25-0.73)/0.73 = 71%). These raw results would suggest that PPM ‘negatively’ impacted in preventing TB deaths, but these estimates do not account for the two factors mentioned in the previous paragraph of our response.

Ultimately, we feel that revisions to our fifth paragraph of the discussion section (starting with “Second, there was”) would adequately address reviewer’s suggestion/concern and appropriately discuss limits of the PPM intervention effect (Lines 459 - 475). We hope this revised text adequately addresses the reviewer’s concern:

“Second, it can be inferred that the South Korean PPM program may not had equal level of impact in improving TB deaths as compared to TS and LTFU. While death is an important outcome indicator to evaluate impact, we find that TS and LTFU more relevant outcomes to assess the policy level impact of the primary objective and function of the PPM (i.e. improving patient’s treatment management and adherence) as estimates of TB deaths are likely more influence by factors such as age and severity of the disease. In South Korea, TB disease burden is higher in the elderly population (can naturally lead to more observation of death regardless of the PPM intervention – Table 2). Moreover, there was a steady decline of TB patients managed in the public sector over the five-year period. With the complete NHI service coverage of all TB-related health services provided in the private sector since 2011, it is likely that patients preferred to be treated in the private sector where service and infrastructure are more comprehensive and superior to those provided in the public sector. Similarly, the private sector generally manages more serious cases of TB. Consequently, it can be inferred that public sector patients may be suffering from less complicated disease and thus may naturally have a better chance for successful treatment outcomes with minimal intervention. Unfortunately, both of these factors (patient’s socioeconomic position, care-seeking preferences, and disease severity) could not be assessed through data that was available for our study.”

Minor comments
a) Page 51, line 152: "formation of global working groups at the Stop TB"
should be "formation of a global PPM working group hosted by the Stop TB Partnership"

🡺 Thank you for your suggestion. We have revised the text accordingly (see line 149):

“formation of global PPM working groups hosted by the Stop TB Partnership.”

b) Page 51, line 159: "As such, there currently lacks long-term evidence of fully scaled-up national PPM program(s)"
Apart from fixing the grammar, this is a defensible statement, and indeed the authors may wish to cite <https://pubmed.ncbi.nlm.nih.gov/30926734/> to support this claim.
So I'm OK with the statement remaining. But in addition it would only be fair to add an acknowledgement of the very significant expansion of PPM in India. India does not have fully national coverage as of yet, and the outcomes are not yet fully clear - hence the authors' claim being defensible. But considering the far, far greater size of both the population and the TB burden in India relative to South Korea, this huge PPM effort in India should at least be acknowledged. I suggest citing the most recent Joint Monitoring Mission for the India national TB program in support of this; it can be found at <https://tbcindia.gov.in/showfile.php?lid=3536> (it has an extensive section on PPM progress).

🡺Thank you for these detailed comments and suggestions. We acknowledge the significant efforts and progress that are currently being made in India to improve private provider engagement in TB care and we have revised relevant texts in our manuscript to reflect this fact and the reference suggested:

Lines 155-159: “India has made significant progress in increasing TB notification and treatment success rates in the private sector by consolidating government efforts to scale-up private provider engagement interventions through PPM (under the National Multisectoral Action Framework for TB-Free India); yet, it has faced a wide range of challenges in implementing, scaling-up the coverage, and data reporting for its PPM program [14].”

c) Page 59, line 340: "More non-Korean citizen, however, sought TB care in the public sector in both years"
I think what you actually mean is: "The proportion of the patients who were non-Korean citizens was higher in the public sector cohort than in the private sector cohort in both years…."

🡺 Thank you for your suggestion. We have revised the text accordingly (Line 347-349):

"The proportion of the patients who were non-Korean citizen was higher in the public sector in both years (5.8% and 8.9% compared to 3.8% in the private sector in 2009 and 2014).”

d) Page 60, line 366: "[people in the private sector and with transfer history…] did not demonstrate to have improvements in TS as a result of the PPM program between 2009 and 2014"
This is incorrect, since these are DID values, not absolute improvement values.
It should read: "For certain patient groups - [over 60, those with transfer history, etc etc] - any improvement in TS for private sector patients was no greater than any improvement in TS for public sector patients, so there was not a completely clear impact from the PPM program between 2009 and 2014."
The same issue comes up on page 62, line 418: "elderly (60 or older) and culture confirmed patients managed in the private sector clearly did not benefit from the PPM program". Not true. They just didn't improve as rapidly as the improvements in the public sector cohort.

🡺 Thank you for these important corrections and detailed feedback. We have revised relevant texts accordingly: 373-377 and 430-432):

Lines 373-377 for the first comment: “For certain patient sub-groups (60 years or older, those with transfer history, and positive culture results), difference in difference estimate was not statistically significant (i.e. 95% confidence interval crossing 0.0), inferring that the PPM program may not have made clear impact on improving TS in these sub-group populations.”

Lines 430-432 for the second comment: “the impact of the PPM program for elderly (60 or older) and culture confirmed TB patients managed in the private sector may not have been as beneficial as the same patient groups managed in the public sector.”

e) Page 62, line 410: "as many high TB burden countries with emerging economies shift towards
411 universal health coverage (UHC),"
This somewhat mixes the concept of national health insurance (NHI, and/or social health insurance (SHI)), which was the topic of the paragraph up to this point, with UHC. Obviously, those two concepts are not the same (NHI is one potential way of trying to achieve UHC). Maybe make that clearer. And consider including the following citation on the topic of TB and SHI/NHI: Wells WA, Stallworthy G, Balsara Z. How tuberculosis programs can navigate the world of social health insurance. Int J Tuberc Lung Dis. 2019. 23:26-37. doi: 10.5588/ijtld.18.0289.

🡺 We absolutely agree with your opinion and revised the text accordingly and cited suggested reference (line 421-423):

“designing and optimizing scale-up plans for PPM programs around various on-going efforts to improve social health insurance coverage (one of several ways to achieve Universal Health Coverage) in high TB burden settings [35]”

f) Page 65, line 485: "to integrate key private sectors" should be "to integrate key private sector providers"

🡺 Thank you for your suggestion. We have revised the text accordingly in lines 504:

“to integrate key private sector providers”
