# Supplementary material for: Evaluating the impact of the nationwide public–private mix (PPM) program for tuberculosis under National Health Insurance in South Korea: A difference in differences analysis
Source: PLoS Med. 2021 Jul 14;18(7):e1003717. doi: 10.1371/journal.pmed.1003717 (PMC8318235; doi:10.1371/journal.pmed.1003717)
Supplement: Attachment — Submitted filename: PMEDICINE-D-19-03769_R3_Response to editor.docx [file pmed.1003717.s008.docx]

**Editorial comments**

At line 53, please make that "... to date.".

Thank you for this suggestion and we’ve revised the text accordingly (to this date 🡪 to date).

Around line 65, please quote summary information on sex and average age for the two cohorts.

Thank you for this suggestion. We’ve inserted summary information on sex and mean age for the respective cohorts in parenthesis in lines between 65 and 68).

We think that an additional short sentence is needed immediately prior to line 70, quoting the percentages for TS in private and public sectors at the two time points (as at line 355).

At line 70, please reword "As a result ..." to avoid making a claim of causality in this observational study, e.g., to "After ..."; and remove the word "vastly".

Thank you for these suggestions. We’ve revised the texts from line 70 onwards to address above two comments as below:

“After expanding nationwide PPM, absolute treatment success rates improved by 9.10% (87.5% to 93.4%) and by 13.3% (from 70.3% to 83.9%) in the public and private sectors respectively. Relative to the public sector, the private sector saw 4.1% (95% Confidence Interval [CI], 2.9 to 5.3% and p-value < 0.001) and - 8.7% (95% CI -9.7 to -7.7%, p-value <0.001) higher rates of improvement in treatment success and reduction in lost to follow-up.”

Please add "in South Korea" around line 80.

We’ve revised the text accordingly.

At line 119, please replace "effectively reduced", implying a causal interpretation, with "... was associated with a reduction" or similar, as appropriate for the observational research design.

We’ve revised the text accordingly.

Please confirm that there were no data-driven changes to the analysis plan.

We confirm that there were no data-driven changes across all of our revisions.

At line 393, for example, please avoid the claim of causality implicit in "... was central in improving ...", and instead state "was associated with an improvement in patient outcomes ..." or similar.

We’ve revised the text accordingly.

Please make similar adaptations to any other elements of text reflecting a causal interpretation.

We have reviewed the text throughout the manuscript and made necessary revisions to avoid any implicit causality statements.

Please remove the word "greatly" at line 396. Again, "... as a result" implies causality and should be amended. 

We’ve revised the text accordingly.

To the sentence beginning at line 397 ("Improvements in treatment outcomes ...") please add the caveat that the outcomes studied remained superior in the public sector. 

At line 459, should that be "... may not have had an equal ..."?

We’ve revised the text accordingly.

Please use the style "... 5 years" throughout the text, although numbers should be written out at the start of sentences. 

We’ve modified the text from “five years” to “5 years” throughout the manuscript.

Throughout the text, please remove spaces from within the reference call-outs, e.g., "... (Figure 1A) [18,19].".

We’ve revised the text accordingly.

In the figures and tables, and any other instances in the ms, please convert "p<0.0001" to "p<0.001".

We’ve converted “p<0.0001” to “p<0.001” throughout the main manuscript and supplementary documents’ figures and tables.

In the first two rows of the STROBE checklist, right hand column, we suggest simply specifying "title" and "abstract" rather than giving the page numbers.

We’ve simplified the STROBE checklist as suggested 🡪 converted the page numbers in the first two rows and the right hand column to “Title & Abstract”.
