# Supplementary material for: Evaluating the impact of the nationwide public–private mix (PPM) program for tuberculosis under National Health Insurance in South Korea: A difference in differences analysis
Source: PLoS Med. 2021 Jul 14;18(7):e1003717. doi: 10.1371/journal.pmed.1003717 (PMC8318235; doi:10.1371/journal.pmed.1003717)
Supplement: Attachment — Submitted filename: PMEDICINE-D-19-03769_R4_Response to editor.docx [file pmed.1003717.s009.docx]

**Requests from Editors:**

In the data statement, you provide a web address for Statistics Korea, which has a "contact" section. Are you able to also provide a specific contact address (institutional email address preferred) at KCDA for readers interested in inquiring about access to study data?

*Queries for data access can be made to Ms. Kim, Jinsun at KCDA (email: lilykim@korea.kr). We’ve included this information data availability statement in the manuscript submission page.*

At line 69 (abstract) the second "private" can be removed.

*We’ve deleted second ‘private’ from the text.*

At line 75, please make that "loss to follow-up".

*We’ve revised the sentence accordingly.*

Please add a new final sentence to the "Methods and findings" subsection of your abstract, which should begin "Study limitations include ..." or similar and should quote 2-3 of the study's main limitations.

*We’ve added following two sentences at the end of the “Methods and finding” subsection of our abstract (line 77-79):*

*“Study limitations include non-longitudinal nature of our original data set, inability to assess the regional disparities and verify PPM program’s impact on TB mortality.”*

At line 117, should that be "reduction in ..."?

*We’ve revised the text accordingly (line 120).*

At line 358, we suggest "indicates" rather than "infers".

*We’ve revised the text accordingly (line 361).*
